# Grief reactions in relation to professional and social support among family members of persons who died from sudden cardiac arrest: A longitudinal survey study

**DOI:** 10.1016/j.resplu.2022.100318

**Published:** 2022-10-19

**Authors:** Nina Carlsson, Anette Alvariza, Lena Axelsson, Anders Bremer, Kristofer Årestedt

**Affiliations:** a Faculty of Health and Life Sciences, Linnaeus University, Kalmar/Växjö, Sweden; b Department of Health Care Sciences/Palliative Research Centre, Marie Cederschiöld University, Stockholm, Sweden; c Capio, Palliative Care, Dalen Hospital, Stockholm, Sweden; d Sophiahemmet University, Stockholm, Sweden; e Department of Ambulance Service, Region Kalmar County, Kalmar, Sweden; f Department of Research, Region Kalmar County, Kalmar, Sweden

**Keywords:** Anxiety, Cardiac arrest, Depression, Posttraumatic stress, Prolonged grief, Social support

## Abstract

**Background:**

The loss of a close person from sudden cardiac arrest (CA) leaves family members at risk of developing grief reactions such as symptoms of prolonged grief, anxiety, depression, and posttraumatic stress. The aim was to describe longitudinal variations in grief reactions and its association with professional and social support among bereaved family members after a close person’s death from sudden CA.

**Methods:**

This longitudinal multimethod survey included 69 bereaved family members who completed a questionnaire 6 and 12-months after the CA, including the Prolonged Grief Disorder-13, Hospital Anxiety and Depression Scale, PTSD Checklist for DSM-5, and Multidimensional Scale of Perceived Social Support. Qualitative data were collected by open-ended questions. Quantitative data was analyzed using Wilcoxon signed-rank test and linear regression analysis while written comments were analyzed using qualitative content analysis.

**Results:**

The median age was 62 years, 67 % were women, and 38 % had been present during the resuscitation attempts. Using the cut-off scores at the 6- and 12-month assessments respectively, 14 % and 17 % reported symptoms of prolonged grief, 32 % and 26 % symptoms of anxiety, 14 % and 9 % depression, and 4 % and 1 % posttraumatic stress. Professional and social support at the 6-month assessment were significantly associated with symptoms of prolonged grief, anxiety, depression, and/or posttraumatic stress at the 12-month assessments but could not predict any changes in the grief reactions.

**Conclusions:**

Family members’ grief reactions point to the importance of proactive and available support over time to meet family members’ needs.

## Introduction

Cardiac arrest (CA) is a severe condition associated with high mortality,^1^, ^2^, ^3^ and this leaves many family members in grief after the sudden loss of a close person. Most people have resilience to cope with such loss,^4^ but it is of importance to recognize that the suddenness of death, can increase the risk of developing complicated grief reactions^5^ such as symptoms of prolonged grief,^6^, ^7^ anxiety,^8^, ^9^ depression,^7^, ^9^ and posttraumatic stress.^7^, ^10^

Family members often have post resuscitation care needs such as organized professional support in terms of information and emotional support.^11^, ^12^ However, studies show that health care professionals lack the competence to adequately meet family members’ reactions at the time of resuscitation attempts and death.^13^, ^14^ Social support from friends and family is also needed and found to be of importance with the potential to reduce the incidence of complicated grief reactions.^15^, ^16^ Even though the conceptualisation of social support varies,^16^, ^17^ it seems closely connected to the possibility to talk with family members and friends about one’s feelings and what has happened. Barriers to optimal social support can thus be connected to problems in communication and to feelings of not being understood.^18^

Although professional and social support is considered important, little is known about its relation to grief reactions in bereaved family members to persons who die from sudden CA. A review study about social support in people bereaved by sudden or violent causes of death concluded that social support is associated with less severe grief reactions.^16^ The review did not include any study about CA, but recent results from our research group shows that both professional and social support were associated with grief reactions such as symptoms of prolonged grief, anxiety, depression, and/or posttraumatic stress 6 months post CA.^19^ However, no study has investigated these associations over longer time periods even though the family members’ support needs are likely to change. Hence, the aim was to describe longitudinal variations in grief reactions and its association with professional and social support among bereaved family members after a close person’s death from sudden CA.

## Methods

### Design

This longitudinal multimethod survey study used both quantitative and qualitative data collected 6 and 12-month post CA. Data was taken from a larger research project about grief reactions among bereaved family members due to death from sudden CA.^19^, ^20^ Data were collected between September 2018 and January 2021. In the present study, only family members who completed the 12-months assessment were included. Approval was granted from the Regional Ethical Review Board in Linköping, Sweden (No. 2017/525–31).

### Sample and procedure

The included family members were 18 years or older and had lost an adult family member to sudden CA, in-hospital or out-of-hospital, where cardiopulmonary resuscitation attempts had been initiated. The CA should also be caused by heart or lung disease. Further, family members had to understand Swedish to be included. Family member was defined according to Whall’s definition as two or more individuals functioning in a way that they perceive as a family. Thus, not necessarily connected by blood ties or by law.^21^

A regional part of the Swedish Register of Cardiopulmonary Resuscitation (https://shlr.registercentrum.se/), in south-eastern Sweden, was screened to identify persons who had died from CA. Deceased persons with a documented Do Not Resuscitate order were not screened. Family members and their contact information were identified using the patient records. They were then contacted by phone for study information by the first author. Family members who were interested to participate received a postal questionnaire to complete at home.

### The questionnaire

Data were collected through a questionnaire that contained questions about the family members’ demographic characteristics, the CA, and professional support from the healthcare services (for example psychologist, counsellor, and/or psychiatrist). The questionnaire also contained self-reported instruments to measure symptoms of prolonged grief, anxiety, depression, posttraumatic stress, and perceived social support. Each instrument was followed by an open-ended question, for example: *‘If you have any reflections or comments about this part of the survey that concerns grief, you are welcome to write them here.* An overview of the included instruments is presented in Table 1.

**Table 1:** Overview of self-reported instruments.

Instruments	Constructs	Items	Scales	Score range	Cronbach’s α a
PG-13	Prolonged grief	13	One total scale	11–55	0.93 (0.90, 0.95)
HADS	Anxiety and depression	14	Two subscales (anxiety and depression)	0–21	0.90 (0.86, 0.93) 0.88 (0.84, 0.92)
PCL-5	Posttraumatic stress	20	One total scale	0–80	0.94 (0.92, 0.96)
MSPSS	Perceived social support	12	One total scale and three subscales (family, friend, and significant others)	1–7	0.93 (0.91, 0.95) 0.88 (0.82, 0.92) 0.95 (0.93, 0.97) 0.94 (0.92, 0.96)

PG-13 = Prolonged Grief Disorder-13, HADS = Hospital Anxiety and Depression Scale, PCL-5 = PTSD Checklist for DSM-5, MSPSS = Multidimensional Scale of Perceived Social Support.

a Cronbach’s alpha values in the present study including the 95% confidence interval within brackets.

#### Prolonged grief Disorder-13 (PG-13)

The PG-13 was used to measure symptoms of prolonged grief.^22^, ^23^ The instrument consists of 13 items, of which two are not used to calculate the total score. The remaining items cover cognitive, behavioural, and emotional symptoms. All items have a five-point response format, ranging from ‘Not at all’ (1) to ‘Overwhelmingly’ (5) or from ‘Not at all’ (1) to ‘Several times a day’ (5). The total score is calculated by summing the responses, and the possible score range is 11 to 55; higher score indicates more symptoms of prolonged grief. A cut-off score of ≥ 35 has been suggested.^22^ The PG-13 has shown satisfactory reliability and validity.^22^, ^24^

#### Hospital anxiety and depression scale (HADS)

The HADS was used to measure symptoms of anxiety and depression.^25^ The HADS consists of 14 items divided into two subscales; symptoms of anxiety (seven items) and symptoms of depression (seven items). Each item has a four-point response format ranging from 0 to 3. The subscale scores are calculated by summing the item responses and the possible score range is 0 to 21; higher scores indicating higher symptom levels. The proposed cut-off scores are as follows: normal range (0–7), suggested presence (8–10), and probable presence (11–21).^26^ The HADS has shown good reliability and validity.^27^, ^28^

#### PTSD Checklist for DSM-5 (PCL-5)

The PCL-5 was used to measure symptoms of posttraumatic stress.^29^ The PCL-5 consists of 20 items with a response format that ranges from ‘Not at all’ (0) to ‘Extremely’ (4). The total scale score is calculated by summing the item responses, and the possible range is 0 to 80. Higher scores indicate higher symptom levels of posttraumatic stress, and a cut-off score of ≥ 38 has been suggested.^29^ The PLC-5 has shown good reliability and validity.^29^, ^30^

#### The Multidimensional scale of perceived social support (MSPSS)

The MSPSS was used to measure perceived social support.^17^ The instrument consists of 12 items that cover support from family (4 items), friends (4 items), and significant others (4 items). All items have a seven-point response format ranging from ‘Very strongly disagree’ (1) to ‘Very strongly agree’ (7). A total score can be calculated by summing the item responses and dividing the sum by the number of items. Thus, the possible score range is between 1 and 7, and higher scores indicate higher levels of perceived social support. Three subscales (family, friends, and significant others) can also be calculated by applying the same scoring. No cut-off scores have been suggested. The MSPSS has shown good reliability and validity.^17^, ^31^

## Statistical analysis

Missing data not exceeding 25 % per scale were replaced using the persons mean score. In total, seven values from seven different participants were replaced. Descriptive statistics were used to present family members’ characteristics. Continuous data are presented as means and standard deviations, ordered categorical data with medians and quartiles, and non-ordered categorical data with frequencies. Wilcoxon rank-sum test was used to compare differences in symptoms of prolonged grief, anxiety, depression, and posttraumatic stress between family members who was present during the resuscitation and those who were not.

The McNemar test was used to investigate changes in the cut-off scores between the 6 and 12-month assessments while the Wilcoxon signed-rank test was used to investigate changes in the scale scores. The effect size for the Wilcoxon signed-rank test was calculated using Cohen’s r (small = 0.02–0.30, medium = 0.30–0.50, and large > 0.50).^32^ In addition, to illustrate the number of family members who changed in the outcome measures (PG-13, HADS and PCL-5) between the two assessments, change scores were calculated by subtracting the 12-month assessment from the 6-month assessment; negative change scores reflects decreased symptom levels and positive change scores increased symptom levels.

Simple linear regression analysis was used to explore the associations between the exploratory variables (professional and social support) from the 6-month assessment and the outcome measures (PG-13, HADS, and PCL-5) at the 12-month assessment. To predict the change between the 6 and 12-month assessment, residualized scores were used as outcome variables. This procedure is commonly recommended to measure change using linear regression analysis and implies that the regression is conducted in two steps. First, the 12-month assessment of the outcome variable is regressed on the 6-month assessment. Second, the residualized scores from step one (the proportion of the outcome variable at the 12-month that is not explained by the 6-month assessment) is used as the outcome variable, regressed on the explanatory variable in a simple linear regression.^33^ Nonparametric bootstrapped confidence intervals and p-values, based on 2000 replications, was calculated for all regression analyses.

The level of significance was set at p < 0.05. The regression analyses were performed using the R 4.2.1 (R Foundation for Statistical Computing, Vienna, Austria), including the boot.pval 0.4, sjmisc 2.8.9, and summarytools 1.0.1 packages.

### Qualitative analysis

To enhance the understanding of grief reactions as well as professional and social support, the responses to the open-ended questions were analysed using a qualitative content analysis.^34^ The analysis was conducted through searching for descriptions and explanations that could complement, illustrate, and further explain the answers that participants had reported in the instruments in the questionnaire. The comments from the open-ended questions were read through several times and then compiled and organized as grief reactions in relation to professional support and social support. Further, the results of the analysis were discussed in steps by the authors to reach consensus and to agree upon the presentation. In total, 49 participants had written 129 comments. The comments varied from a few words to full pages about thoughts and emotions.

## Results

### Participants flow and characteristics of participants

**Fig. 1:**
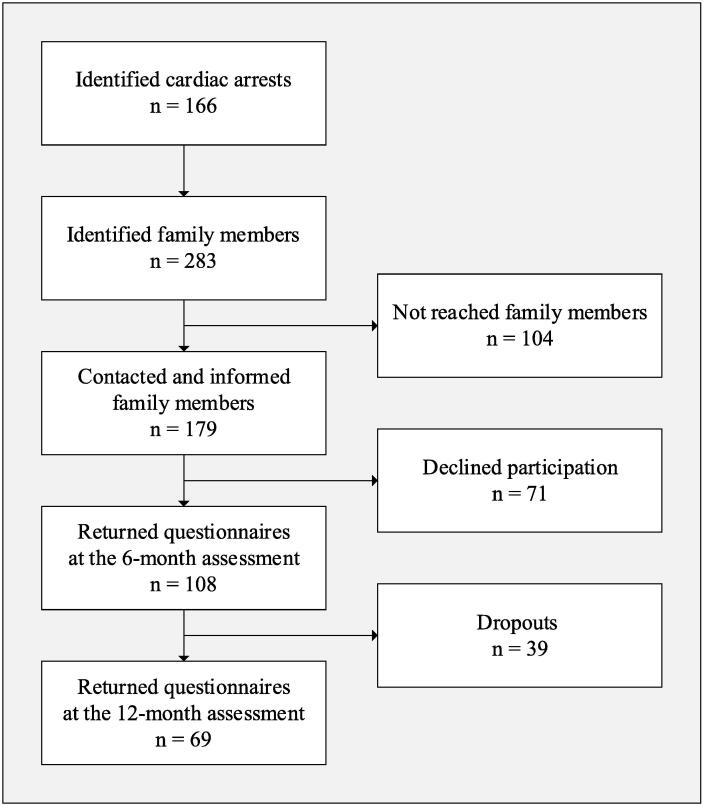
Flowchart over the inclusion of family members 6 and 12 months after the death.

During the study period 166 CA events were identified. Of 283 identified family members, 179 were contacted for information and inclusion. In total, 108 questionnaires were returned for the 6-month assessment. The follow-up assessment for the present study was conducted 12 months after the death and was completed by 69 family members (Fig. 1). No significant differences were shown between the participants and dropouts regarding sex, age, prolonged grief, anxiety, depression, or posttraumatic stress.

The participants in the present study were family members of 52 deceased persons 60–91 years old, of whom 56 % (n = 29) were men. The median age of the participants was 62 years (IQR = 52–71), a majority were women (n = 46, 67 %). About one third of the participants (n = 26, 38 %) had been present during the resuscitation attempts and they reported significantly higher symptom levels of prolonged grief 6 months after the death (z = -2.53, p = 0.011) and higher symptom levels of posttraumatic stress at both 6 and 12 months after the death (z = -2.65, p = 0.008 and z = -2.16, p = 0.031) compared to those who was not present. More details about the participants are presented in Table 2.

**Table 2:** Characteristics of participants (n = 69).

Age, Mdn (IQR)	62 (52–71)
Sex, n (%)	
Woman	46 (67)
Men	23 (33)
Country of birth, n (%)	
Sweden	67 (97)
Other Nordic country	2 (3)
Marital status, n (%)	
Widow/widower	27 (39)
Married/registered partner	25 (36)
Unmarried	11 (16)
Divorced	6 (9)
Cohabiting, n (%)	
Living alone	33 (48)
Living with someone else	36 (52)
Occupation, n (%)	
Employed	29 (42)
Unemployed	1 (1)
Retired	34 (49)
Sick leave	4 (6)
Student	1 (1)
Highest education, n (%)	
Primary school	17 (25)
High school	26 (39)
University	24 (36)
Missing data	2
Relation to the deceased, n (%)	
Spouse	25 (36)
Adult child	36 (52)
Parent	1 (1)
Sibling	3 (4)
Father-in-law	1 (1)
Daughter-in-law	1 (1)
Nephews	1 (1)
Friend	1 (1)
Been present duringresuscitation attempts, n (%)	26 (38)
Professional support, n (%)	20 (29)
Social support 6 months post death, Mdn (IQR)	
MSPSS total scale	6.4 (5.4–6.9)
Family	6.8 (5.5–7.0)
Friends	6.0 (5.0–7.0)
Significant others	6.8 (5.5–7.0)

MSPSS = Multidimensional Scale of Perceived Social Support.

### Variations in symptoms of prolonged grief, anxiety, depression, and posttraumatic stress

Analyses of the cut-off scores at the 6-month assessment showed that symptom of anxiety was most common (n = 22, 32 %) followed by prolonged grief (n = 10, 14 %) and depression (n = 10, 14 %) and finally posttraumatic stress (n = 3, 4 %). At the 12-month assessment, the same pattern was seen, but prolonged grief was now more common than depression (17 % and 9 %, respectively). Based on these cut-off scores, no significant changes were shown from the 6-month assessment to the 12-month assessment (Table 3).

**Table 3:** Symptoms of prolonged grief, posttraumatic stress, anxiety, and depression 6 and 12 months after death (n = 69).

	6-month assessment	12-month assessment	p-value	Effect size c
Symptoms of prolonged grief (PG-13), Mdn (IQR)	22 (16–32)	21 (16–28)	0.013 a	0.29
No cases (11–34), n (%)	59 (86)	57 (83)	0.724^b^	
Cases (35–55), n (%)	10 (14)	12 (17)		
Symptoms of anxiety, (HADS), Mdn (IQR)	5 (2–9)	4 (1–8)	0.016 a	0.28
Normal range (0–7), n (%)	47 (68)	51 (74)	0.387^b^	
Suggested or probable presence (8–21), n (%)	22 (32)	18 (26)		
Symptoms of depression, (HADS), Mdn (IQR)	2 (1–6)	2 (1–4)	0.142 a	0.17
Normal range (0–7), n (%)	59 (86)	63 (91)	0.206^b^	
Suggested or probable presence (8–21), n (%)	10 (14)	6 (9)		
Symptoms of posttraumatic stress (PCL-5), Mdn (IQR)	7 (2–19)	8 (2–16)	0.677 a	0.07
No cases (0–37), n (%)	66 (96)	68 (99)	0.480^b^	
Cases (38–80), n (%)	3 (4)	1 (1)		

PG-13 = Prolonged Grief Disorder-13, HADS = Hospital Anxiety and Depression Scale, PCL-5 = PTSD Checklist for DSM-5.

a Wilcoxon signed-rank test with continuity correction.

b McNemar test with continuity correction.

c Cohen’s r (small = 0.02–0.30, medium = 0.30–0.50, large > 0.50).

Analyses of the scale scores showed a significant decrease in symptoms of prolonged grief (p = 0.017) and anxiety (p = 0.021) between the two assessments but the effect size was small, 0.29 and 0.28 respectively. No significant change was seen for symptoms of depression and posttraumatic stress (Table 3).

**Fig. 2:**
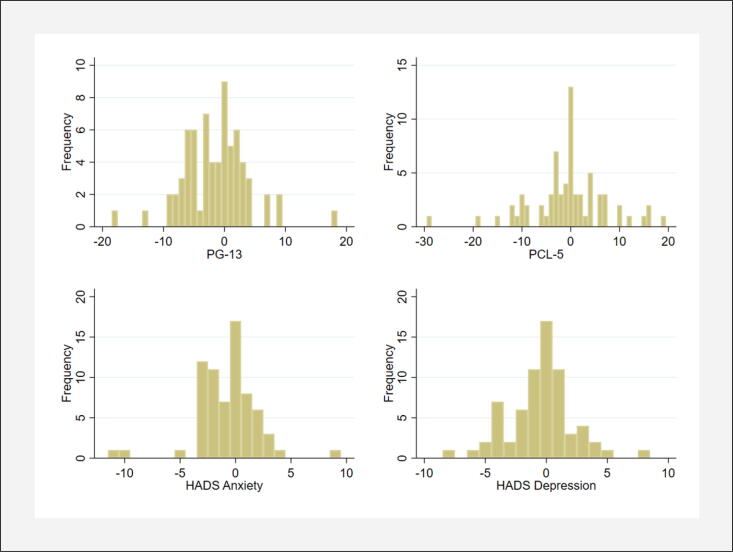
The graph illustrates the distribution of change scores between the 6-month assessment and the 12-month assessment for Prolonged Grief Disorder-13 (PG-13), Hospital Anxiety and Depression Scale (HADS), and PTSD Checklist for DSM-5 (PCL-5). Zero values imply unchanged symptom levels while negative change scores reflect decreased symptom levels and positive values reflect higher symptom levels at the 12-month assessment compared to the 6-month assessment.

According to the change scores, most bereaved family members improved or did not change in symptoms of prolonged grief, anxiety, depression, and posttraumatic stress between the 6 and 12-month assessment. However, a substantial share reported higher symptom levels at the 12-month assessment compared to the 6-month assessment (Fig. 2). Increased symptom levels were most common for posttraumatic stress (n = 25, 36 %) followed by prolonged grief (n = 23, 33 %), depression (n = 22, 32 %), and anxiety (n = 17, 25 %).

### Grief reactions in relation to professional support

Professional support at the 6-month assessment was significantly associated with symptoms of prolonged grief, anxiety, depression, and posttraumatic stress at the 12-month assessment. Hence, having professional support implied higher symptom levels. Based on the residualized scores, professional support could not predict the change in any of the outcome variables (Table 4).

**Table 4:** Prolonged grief, anxiety, depression, and post-traumatic stress 6 months after death in relation to professional and social support 12 months after death, based on simple linear regression analyses.

Outcome variables	Explanatory variables	B (CI95%) a 12-month assessment	R^2^	B (CI95%) a Residualized change scores	R^2^
PG-13	Professional support	8.87 (4.22, 13.59) ***	0.16	0.60 (−1.91, 3.18)	<0.01
	MSPSS total scale	−1.99 (−4.38, 0.25)	0.04	1.11 (−0.07, 2.25)	0.05
	Family	−1.72 (−4.08, 0.68)	0.03	0.99 (−0.24, 2.13)	0.04
	Friends	−1.11 (−2.96, 0.78)	0.02	0.91 (−0.07, 1.86)	0.05
	Significant others	−1.39 (−3.15, 0.14)	0.04	0.56 (−0.38, 1.35)	0.03
HADS anxiety	Professional support	2.69 (0.71, 4.87) **	0.09	0.74 (−0.54, 2.08)	0.02
	MSPSS total scale	−0.95 (−1.93, 0.02)	0.06	0.27 (−0.31, 0.85)	0.01
	Family	−0.93 (−1.91, 0.06)	0.05	0.25 (−0.35, 0.84)	0.01
	Friends	−0.94 (−1.71, −0.19) *	0.08	0.22 (−0.26, 0.69)	0.01
	Significant others	−0.31 (−1.06, 0.34)	0.01	0.13 (−0.30, 0.56)	0.01
HADS depression	Professional support	1.91 (0.45, 3.46) *	0.08	0.17 (−1.02, 1.39)	<0.01
	MSPSS total scale	−1.23 (−1.92, −0.61) **	0.17	−0.18 (−0.74, 0.34)	0.01
	Family	−1.13 (−1.83, −0.48) **	0.14	−0.01 (−0.56, 0.52)	<0.01
	Friends	−0.79 (−1.38, −0.23) **	0.10	0.03 (−0.42, 0.44)	<0.01
	Significant others	−0.75 (−1.29, −0.30) **	0.12	−0.29 (−0.69, 0.04)	0.03
PCL-5	Professional support	8.60 (3.07, 14.88) **	0.11	2.03 (−1.73, 5.87)	0.02
	MSPSS total scale	−4.40 (−7.15, −1.80) **	0.15	−0.34 (−2.05, 1.32)	<0.01
	Family	−4.16 (−7.03, −1.41) **	0.13	−0.25 (−2.04, 1.46)	<0.01
	Friends	−3.19 (−5.40, −0.94) **	0.11	−0.06 (−1.56, 1.36)	<0.01
	Significant others	−2.37 (−4.54, −0.58) **	0.08	−0.35 (−1.61, 0.81)	<0.01

*p < 0.05, **p < 0.01, ***p < 0.001.

PG-13 = Prolonged Grief Disorder-13, HADS = Hospital Anxiety and Depression Scale, PCL-5 = PTSD Checklist for DSM-5, MSPSS = Multidimensional Scale of Perceived Social Support.

a Nonparametric bootstrapped confidence interval, based on 2000 replications.

The family members described how they wanted a peaceful environment and professionals who stayed close and were available for questions and conversations. A daughter commented: *“We were treated wonderfully by the emergency room support staff who were nearby throughout our stay there.”*

Many pondered if they could have acted differently before and during the CA and wrote about self-blame. Follow-up conversations with professionals reduced questions. A daughter commented: *“I’m glad we were told by a doctor, via a later call, about how a sudden cardiac arrest happens. /…/ It helped me even though it was a shock when we got the phone call.”*

A widow commented the delivering of the death notification by phone: *“It was a shock. We weren’t prepared for it, neither me nor my children. I’ve received no support at all from the department [at the hospital] where my husband died. I’ve had to find a doctor and a psychologist myself.”*

Family members wrote about their need for professional support, but lack of time and energy hindered them from seeking the support, and some also had worries about not being able to afford it. A widower wrote about the disappointing lack of support from the health care services, and instead he received social support from colleagues.

### Grief reactions in relation to social support

All sources of social support from the 6-month assessment were significantly associated with symptoms of depression and posttraumatic stress at the 12-month assessment. However, no source of social support from the 6-month assessment was significantly associated with prolonged grief at the 12-month assessment, and only social support from friends was significantly associated with symptoms of anxiety. Based on the residualized scores, social support could not predict the change in any of the outcome variables (Table 4).

The comments revealed that family members often had lost an important part of their social support, the part the deceased person used to provide. A widower commented that the close relation to his family could not ease his longing for his wife. Despite having close relations, some family members wrote about holding back their own grief reactions. One family member commented: *“I struggle with my grief among others. I don’t want to be a burden.”* Family members also worried about other persons’ grief reactions and need of support. A daughter commented: *“The hardest part when my mom died was taking care of my dad. I didn’t have time to grieve, but now I feel better.”* However, family members also wrote about how they engaged in new activities and relationships and thus tried to strengthen their social network. A family member commented that sharing experiences with other bereaved persons in a support group had helped her in her grief process.

## Discussion

To the best of our knowledge, this is one of the first studies that has explored family members’ grief reactions in relation to professional and social support over a period up to one year after a close person’s death from sudden CA. A substantial share of the family members reported higher symptom levels of prolonged grief, anxiety, depression, and posttraumatic stress at the 12-month assessment compared to the 6-month assessment. Both professional and social support were significantly associated with symptoms of prolonged grief, anxiety, depression, and/or posttraumatic stress at the 12-month assessments. In contrast, neither professional support nor social support predicted changes in the grief reactions.

Despite the observation that professional support was associated with higher symptom levels (that is, those who were in greatest need received professional support), several family members in the present study stated that they did not receive the professional support they needed from the health care services. For support to actually be perceived as supportive, it must be available and match the receiving person’s needs.^35^ Emotional and psychological professional support during resuscitation has been shown to reduce symptoms of phycological distress among family members.^36^ For example, having a support person present during resuscitation can be beneficial for both family members and staff as it may reduce stress and anxiety.^37^ This was also stated in the comments by family members in the present study.

Present findings indicate that professional support needs to be proactive. Also, Merlevde et al.^38^ has pointed out that there are gaps in the professional support when death occurs at home and suggests proactive actions, for example providing follow-up measures with a condolence letter including contact information. Even though a follow-up call is often experienced as being helpful in the bereavement process, it can also involve disappointments when family members do not receive the information they wish for, for example, the circumstances concerning the death.^39^ To better address family members’ needs for support, it is essential to educate health care professionals in end-of-life care and how to communicate with family members during and after resuscitation attempts.^36^, ^40^ To identify those in need of further interventions, grief reactions need to be screened, and then matched with appropriate support.^5^, ^15^

Social support seems important after deaths from sudden CA as present findings show associations with prolonged grief, anxiety, depression, and posttraumatic stress. Even though support from family showed the strongest associations, support from friends and other significant persons, such as colleagues, constitute important parts of social networks. Sharing experiences with others was perceived as helpful. Different sources of social support involve different expectations; while family is “supposed” to provide support, support from friends might be judged more positively because it is provided without obligations. Persons with coping abilities might be able to use their social network to cope. However, distressed persons who lack the ability to cope might not be able to request support, and over time drive away potential support.^41^ Peer-support may strengthen social networks and prevent social isolation and loneliness among bereaved persons.^42^ Isolation is known to serve as a stressor contributing to health problems, and social support might alleviate the impact of such stress. However, the support must match the persons’ needs to be supportive, and the challenge in knowing how to deliver support remains.^43^

Many family members were present during the resuscitation attempts. There is substantial evidence supporting family presence during resuscitation.^12^, ^40^ Nevertheless, family presence has been associated with symptoms of anxiety.^19^, ^44^ In the present study, no differences in anxiety were shown. Instead, family presence was associated with higher symptom levels of prolonged grief and posttraumatic stress. It is likely that family presence is of different meanings for different persons and that other factors such as the relation to the deceased person are of greater importance. This explanation is supported by the family members’ comments showing that both being present and not being present at the time of death caused challenges in the grief process. Thus, family members’ individual grief reactions and the ability for coping with bereavement require different kinds of proactive interventions of support after deaths from sudden cardiac arrests.

### Limitations

This study had a limited sample size and hence the results should be generalized with caution. Problems with recruiting participants and high rates of dropouts are common in bereavement research.^38^, ^45^, ^46^ However, the dropout analysis showed no significant differences between participants and non-participants. No reminders were sent out of ethical considerations for bereaved family members’ vulnerable situation. To strengthen the validity, the representative quotations were derived from different participants and will help readers to assess the authenticity and trustworthiness of the results.

## Conclusion

The high symptom levels of grief reactions among family members indicate a need for both professional and social support. Thus, the results point to the importance of proactive and available support over time to meet family members’ various needs.

## Funding

The research was funded by Linnaeus University.

## CRediT authorship contribution statement

**Nina Carlsson:** Conceptualization, Methodology, Data curation, Formal analysis, Writing – original draft. **Anette Alvariza:** Supervision, Conceptualization, Methodology, Writing – review & editing. **Lena Axelsson:** Supervision, Conceptualization. **Anders Bremer:** Supervision, Conceptualization. **Kristofer Årestedt:** Supervision, Conceptualization, Methodology, Data curation, Formal analysis, Writing – review & editing.
